# Artemisinin Prevents Glutamate-Induced Neuronal Cell Death Via Akt Pathway Activation

**DOI:** 10.3389/fncel.2018.00108

**Published:** 2018-04-20

**Authors:** Shao-Peng Lin, Wenjun Li, Ali Winters, Ran Liu, Shao-Hua Yang

**Affiliations:** ^1^Department of Pharmacology and Neuroscience, University of North Texas Health Science Center, Fort Worth, TX, United States; ^2^Department of Emergency, The Second Affiliated Hospital of Guangzhou Medical University, Guangzhou, China

**Keywords:** artemisinin, Akt, oxidative stress, apoptosis, neuroprotection

## Abstract

Artemisinin is an anti-malarial drug that has been in use for almost half century. Recently, novel biological effects of artemisinin on cancer, inflammation-related disorders and cardiovascular disease were reported. However, neuroprotective actions of artemisinin against glutamate-induced oxidative stress have not been investigated. In the current study, we determined the effect of artemisinin against oxidative insult in HT-22 mouse hippocampal cell line. We found that pretreatment of artemisinin declined reactive oxygen species (ROS) production, attenuated the collapse of mitochondrial membrane potential induced by glutamate and rescued HT-22 cells from glutamate-induced cell death. Furthermore, our study demonstrated that artemisinin activated Akt/Bcl-2 signaling and that neuroprotective effect of artemisinin was blocked by Akt-specific inhibitor, MK2206. Taken together, our study indicated that artemisinin prevented neuronal HT-22 cell from glutamate-induced oxidative injury by activation of Akt signaling pathway.

## Introduction

Artemisinin, first discovered in 1970s, has pioneered a new era for the treatment of malaria and saved millions malarial patients worldwide (Guo, 2016). Although the precise mechanism underlying its anti-malaria effect is still not clear, artemisinin and its derivatives are considered prodrugs that generate carbon-centered free radicals or reactive oxygen species (ROS), which further alkylate key parasite proteins and results in the death of parasite (Cui and Su, 2009; Ismail et al., 2016). Furthermore, artemisinin has been indicated to interact with FADH and/or other parasite flavoenzymes, hence impair parasite redox homeostasis and generation of ROS (Haynes et al., 2010). Paradoxically, antioxidant activity of artemisinin has also been demonstrated (Kim et al., 2015). Artemisinin has been found to protect retinal neuronal cells against oxidative stress (Yan et al., 2017).

Oxidative stress refers to the imbalance between ROS production and antioxidant defense which has been found to be involved in aging and aging-related neurodegenerative disorders (Lin et al., 2016; Sozen and Ozer, 2017; Vida et al., 2017). Correspondently, antioxidant has been an attractive approach for the treatment of neurodegenerative diseases (Xie et al., 2013; Lin et al., 2016). Artemisinin could cross the blood-brain barrier (BBB) without obvious toxicity in the central nervous system, implying favorable advantages in the treatment of neurological disorders (Zuo et al., 2016). However, the effect of artemisinin on oxidative stress in brain cells has not been fully investigated. In the present study, we determined the neuroprotective effect of artemisinin on glutamate-induced oxidative injury in HT-22 hippocampal cell line. Our results indicated that artemisinin could prevent neuronal HT-22 cell from glutamate-induced oxidative damage potentially via the activation of Akt pathway.

## Experimental Procedures

### Cell Culture

HT-22 cells, a hippocampal cell line, were maintained in Dulbecco’s Modified Eagle’s Medium (DMEM; HyClone, USA) supplemented with 10% fetal calf serum (FBS; HyClone, USA), 50 IU/ml penicillin and 50 μg/ml streptomycin (Sigma-Aldrich, USA) in a humidified incubator with 5% CO_2_ at 37°C. Cells at passage 10–20 were adjusted to 3 × 10^4^/ml and were plated in 12-well or 96-well cell culture plates (Cellstar, Greiner Bio-One GmbH). At 24 h after seeding, adherent cells were used for all of the experiments. L-Glutamic acid (CAS No. 138-15-8, Sigma-Aldrich, USA) and artemisinin (CAS No. 63968-64-9, Sigma-Aldrich, USA) were purchased from Sigma Aldrich. MK2206 (CAS No. 1032350-13-2) was purchased from Selleck (Houston, TX, USA).

### Cell Viability Assay

Cell viability was assessed using Calcein-AM assay (Anaspec, Fremont, CA, USA) according a protocol modified from our previous publication (Ryou et al., 2015). In brief, cells were washed with phosphate-buffered saline (PBS, pH 7.0) and incubated with 1 μg/ml Calcein-AM for 10 min at 37°C. Fluorescence was determined using a Tecan Infinite F200 plate reader (Maennedorf, Switzerland) with 485/530-nm excitation/emission. The percentage of cell viability was normalized to the control group.

For Calcein-AM/PI double staining, HT-22 cells were incubated in PBS containing 1 μg/ml Calcein-AM and 5 μg/ml propidium iodide (PI, BD Biosciences) at 37°C for 15 min. After washing with PBS, cells were observed by a fluorescence microscope (Axio Observer Z1; Carl Zeiss AG, Germany).

For flow cytometry analysis, floating cells and adherent cells were collected and stained with PI and Annexin V (BD Biosciences) according to the manufacture’s instruction. And the cells were analyzed by a BD flow cytometry (BD Biosciences).

### Reactive Oxygen Species Measurements

Intracellular and mitochondrial ROS production were assessed by a fluorometric assay using 2′,7′-dichlorofluorescein diacetate (H2DCFDA; Invitrogen, USA) and MitoTracker Red CMXRos (Invitrogen, USA), respectively. After 12 h treatment of artemisinin and followed by 12 h treatment of glutamate, cells were incubated in 10 μmol/L H2DCFDA or 0.25 μmol/L MitoTracker Red CMXRos for 30 min at 37°C. The fluorescence was then observed via a fluorescence microscope. The fluorescence was detected with 530/485-nm and 579/599-nm excitation/emission wave lengths.

### Mitochondrial Membrane Potential (ΔΨm) Measurement

The mitochondrial membrane potential was detected using Tetramethylrhodamine, Ethyl Ester (TMRE) mitochondrial membrane potential assay kit (Abcam, USA). Cells were loaded with 20 nM of TMRE working solution for 20 min at 37°C. The fluorescent images were observed and obtained on a Zeiss fluorescence microscope. Fluorescence intensity was measured using a Tecan Infinite F200 plate reader (Maennedorf, Switzerland) with 594/575-nm excitation/emission.

### Immunocytochemistry and TUNEL Staining

Cells were fixed in BD Cytofix/Cytoperm solution (BD Biosciences) and permeabilized using 0.1% Triton-X. The cells were incubated overnight in primary antibody for pAkt (Cell signaling technology, 1:50) followed by staining with Alexa Fluor 488-conjugated goat anti-rabbit IgG (Thermo Scientific, 1:500). Then the cells were further incubated with 0.5 mg/mL DAPI for nuclei. Images were obtained using a LSM 410 confocal microscope (Zeiss, Thornwood, NY, USA).

The fragmentation of genomic DNA was detected by *in situ* staining of DNA ends with TdT-mediated dUTP nick end labeling (TUNEL; Progega, USA) following the manufacturer’s instruction. Briefly, incubation buffer containing Equlibration Buffer, Nucleotide Mix and rTdT Enzyme was incubated for 2 h at 37°C in the dark. Hoechst 33342 staining was used to count the total number of nuclei. Images were taken with a fluorescence microscope.

### Western Blot Analysis

Western blot analysis was carried out using a protocol modified from our previous publication (Xie et al., 2013). In briefly, cell lysate were prepared by homogenization in RIPA buffer (20 mM Tris-HCl, pH 7.5, 150 mM NaCl, 1 mM Na2EDTA, 1 mM EGTA, 1% NP-40 and 1% sodium deoxycholate) including phosphatase and protease inhibitor for 20 min on ice. Proteins were loaded onto 8%–12% SDS-PAGE gel and electrophoresis was performed. Protein samples were transferred to nitrocellulose membranes and incubated with primary antibody overnight at 4°C for Phospho-Akt (Ser473; D9E) XP^®^ Rabbit mAb (Cell signaling technology, 1:2000), Akt (pan; C67E7) Rabbit mAb (Cell signaling technology, 1:1000), Bcl-2 (Cell signaling technology, 1:1000), Bax (Cell signaling technology, 1:1000), Caspase-3 (Cell signaling technology, 1:1000), PARP (Cell signaling technology, 1:1000) and β-Actin (C4; Santa Cruz Biotech, 1:2000) antibody. After being washed three times with PBST, membranes were incubated with horseradish peroxidase-conjugated secondary antibodies for 1 h at room temperature. Membranes were developed with Super Signal West Pico Chemiluminescent Substrate (Thermo Scientific, USA). The optical density of the target protein bands were measured using a Biospectrum 500 imaging system (Ultraviolet Products, Upland, CA, USA).

### Statistical Analysis

Graph Pad Prism 5 was used for statistical analysis. The experiments were carried out at least in triplicate, and all data were presented as mean ± standard error of mean (SEM). *T*-test was used to identify any significant difference between two groups. For comparison of multiple groups, one-way analysis of variance was used and *post hoc* Bonferroni analysis was done to identify the significant differences. For all tests, *p*-value of less than 0.05 was considered significant.

## Results

### Artemisinin Pretreatment Reduced Glutamate-Induced Cytotoxicity in HT-22 Cells

**Figure 1:**
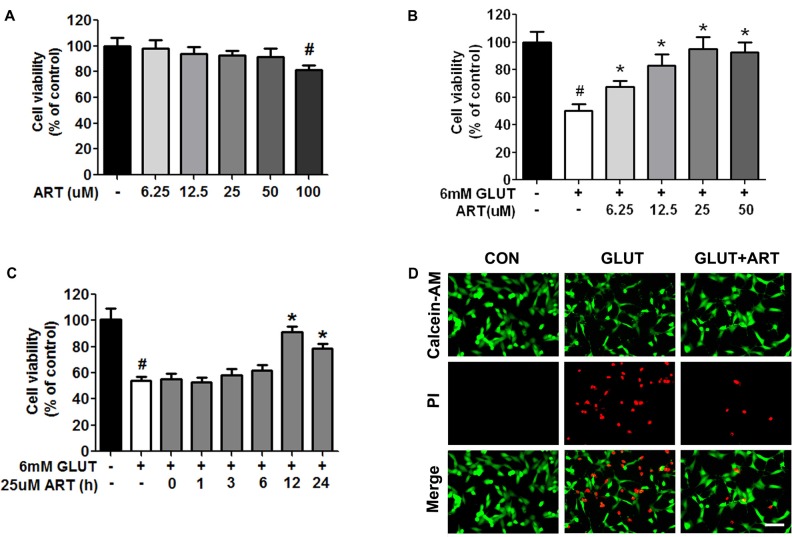
Pretreatment with artemisinin protected HT-22 cells against glutamate-induced cell death. **(A)** Artemisinin concentration between 6.25–50 μM did not cause any cytotoxicity in HT-22 cells. **(B)** Pretreatment with artemisinin for 12 h significantly attenuated glutamate-induced HT-22 cell death in a dose-dependent manner. **(C)** No protective effect against glutamate-induced toxicity was observed when 25 μM artemisinin was pretreated less than 12 h. **(D)** Calcein-AM/PI double staining showed that pretreatment with 25 μM artemisinin for 12 h attenuated glutamate-induced HT-22 cell death. Scale bar = 100 μm. ^#^*p* < 0.05 vs. CON group, *p < 0.05 vs. GLUT group.

HT-22 cells were treated with various concentrations of artemisinin for 24 h and cell viability was analyzed by Calcein-AM assay. No cytotoxic effect was observed upon artemisinin treatment in HT-22 cells until reaching 100 μM concentration (Figure 1A). The effects of artemisinin on glutamate-induced oxidative stress were evaluated in different treatment paradigms. A dose-dependent neuroprotective effect was indicated when HT-22 cells were pretreated with artemisinin for 12 h before 12-h glutamate insult (Figures 1B,D). No protective effect against glutamate toxicity was observed when artemisinin was administered less than 12 h before glutamate insult (Figure 1C). Pretreatment with 25 μM artemisinin for 12 h had an optimum protective effect.

**Figure 2:**
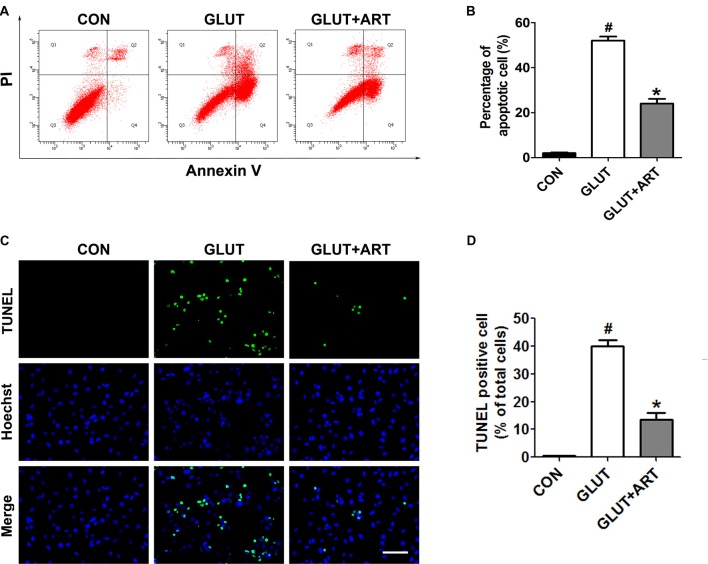
Artemisinin inhibited the glutamate-induced apoptosis in HT-22 cells. **(A,B)** Flow cytometry analysis indicated the anti-apoptotic effect of artemisinin. **(C,D)** TUNEL assay manifested that artemisinin attenuated glutamate-induced cell apoptosis significantly. Scale bar = 100 μm. ^#^*p* < 0.05 vs. CON group, *p < 0.05 vs. GLUT group.

The neuroprotective effect of artemisinin on glutamate insult was further verified by flow cytometry and TUNEL staining. As predicted, glutamate induced a significant increase of apoptosis, which was attenuated by pretreatment of artemisinin (Figure 2).

### Protective Effects of Artemisinin on Glutamate-Induced Oxidative Stress and Loss of Mitochondria Membrane Potential

**Figure 3:**
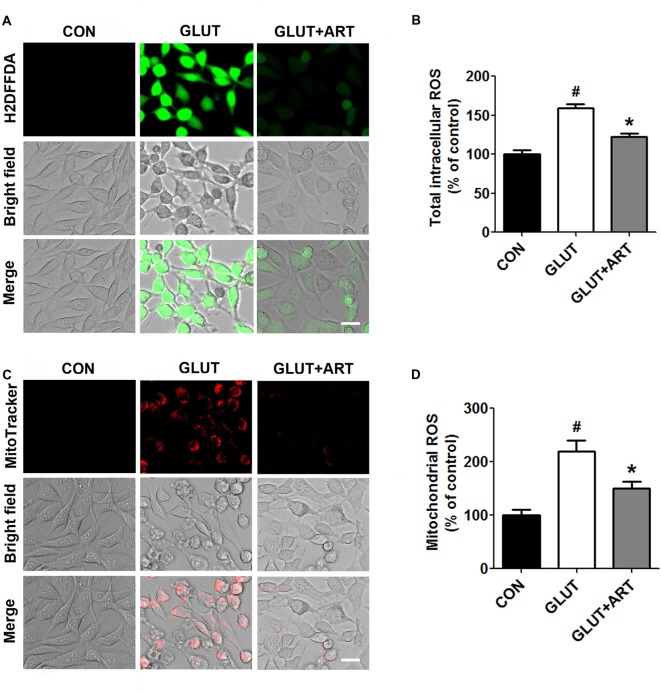
Artemisinin decreased glutamate-induced oxidative stress in HT-22 cells. **(A,B)** Artemisinin reduced the increase of glutamate-induced total intracellular reactive oxygen species (ROS). **(C,D)** Artemisinin decreased the elevation of glutamate-induced mitochondrial ROS. Scale bar = 20 μm. ^#^*p* < 0.05 vs. CON group, *p < 0.05 vs. GLUT group.

**Figure 4:**
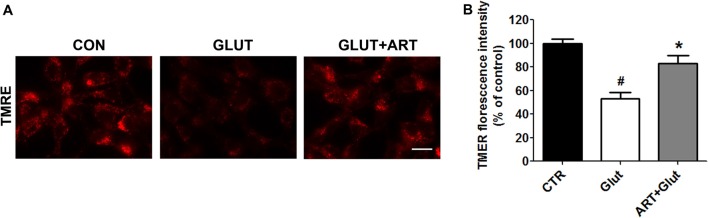
Artemisinin attenuated the glutamate-induced collapse of mitochondrial membrane potential. **(A)** Tetramethylrhodamine, Ethyl Ester (TMRE) staining showed that artemisinin could reduce glutamate-induced ΔΨm loss. **(B)** Quantitative data of **(A)**. Scale bar = 20 μm. ^#^*p* < 0.05 vs. CON group, *p < 0.05 vs. GLUT group.

We determined the effect of artemisinin on glutamate-induced intracellular ROS and mitochondrial ROS production using H2DFFDA and MitoTracker Red CMXRos, respectively. Glutamate significantly increased intracellular ROS and mitochondrial ROS production as compared with control group, which was attenuated upon pretreatment of artemisinin (Figure 3). We further determined the effect of artemisinin pretreatment on mitochondria membrane potential collapse induced by glutamate insult. As predicted, artemisinin reversed glutamate-induced loss of mitochondrial membrane potential evidenced by TMRE analysis (Figure 4).

### Akt Signaling Was Involved in the Neuroprotection of Artemisinin

**Figure 5:**
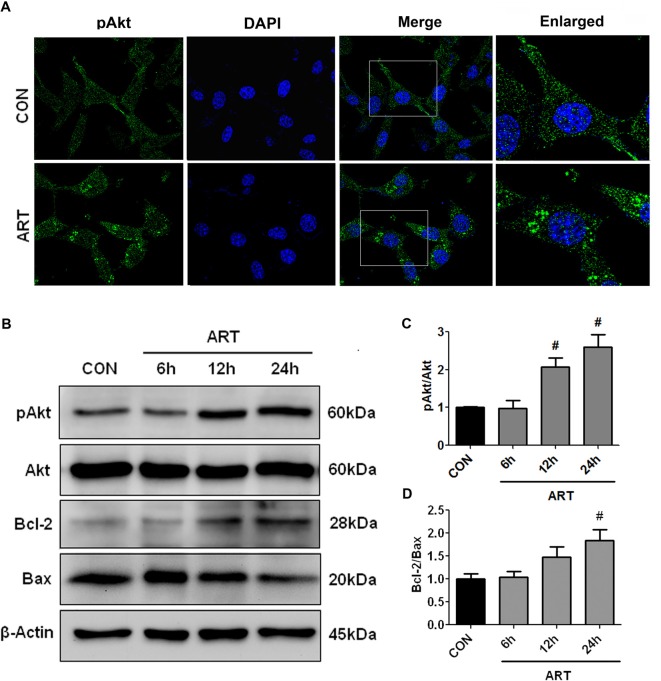
Treatment with artemisinin activated Akt pathway in HT-22 cells. **(A)** Immunocytochemical staining of phosphor-Akt showed that treatment with artemisinin increased Akt phosphorylation. **(B–D)** Western blots demonstrated that artemisinin increased pAkt/Akt and Bcl-2/Bax ratio in a time-dependent manner. ^#^*p* < 0.05 vs. CON group.

To determine whether Akt anti-apoptotic pathway was regulated by artemisinin in HT-22 cells, HT-22 cells were incubated with 25 μM artemisinin for 24 h and then processed for the immunocytochemistry of phosphorylated Akt. An increase of phospho-Akt was observed after 24-h treatment of 25 μM artemisinin (Figure 5A). Consistently, a time-dependent increase of pAkt/Akt and Bcl-2/Bax ratio upon artemisinin treatment was observed by Western Blot (Figures 5B–D).

**Figure 6:**
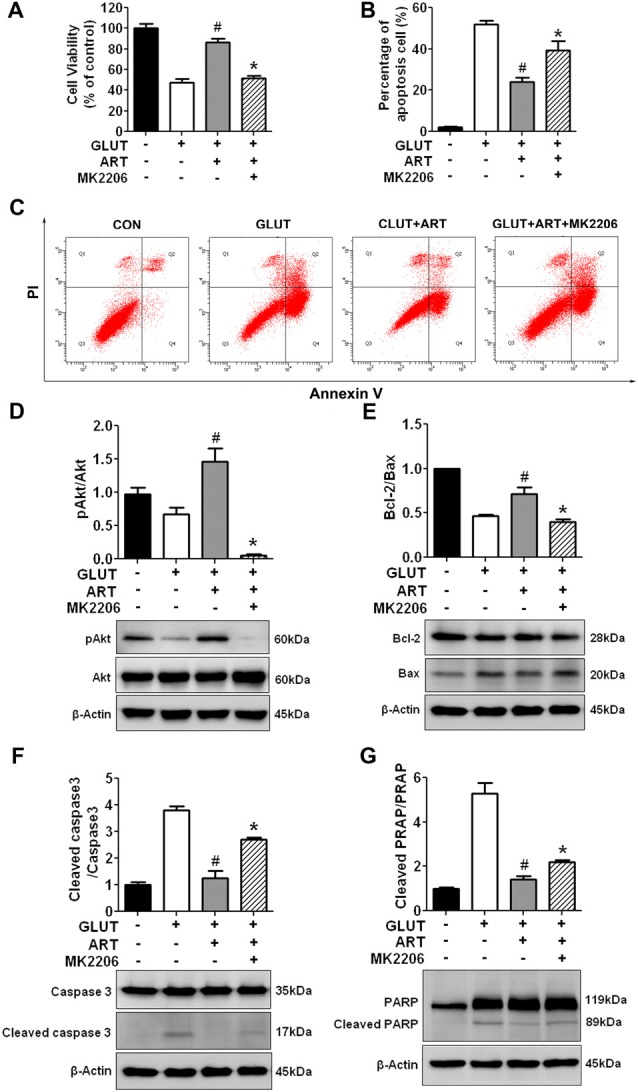
Akt pathway inhibitor MK2206 abolished the protective effect of artemisinin against glutamate insult in HT-22 cells. **(A)** Calcein-AM cell viability assay demonstrated that MK2206 attenuated the protective effects of artemisinin against glutamate-induced cell death. **(B,C)** Annexin V/PI flow cytometry analysis showed that MK2206 abolished the protective effect of artemisinin against glutamate-induced apoptosis. **(D,E)** Western blots analysis indicated that MK2206 attenuated artemisinin-induced increased of pAkt/Akt and Bcl-2/Bax ratio. **(F,G)** Western blots demonstrated that MK2206 abolished artemisinin-induced decrease of cleaved caspase-3 and cleaved PRAP expression in HT-22 cells. The antibodies in Figures 6E,F were incubated in the same gel. Therefore, they had the same reference. ^#^*p* < 0.05 vs. CLUT group, *p < 0.05 vs. GLUT+ART group.

We further determined whether neuroprotective effect of artemisinin was mediated through Akt signaling. Protective effect of artemisinin on glutamate-induced cell death and apoptosis was negated by co-treatment of Akt inhibitor, 5 μM MK2206 (Figures 6A–C). Western blot analysis confirmed that artemisinin-induced increase of pAkt/Akt and Bcl-2/Bax ratio was indeed inhibited with 5 μM MK2206 (Figures 6D,E). In addition, MK2206 abolished artemisinin-induced decrease of cleaved caspase-3 and cleaved PARP (Figures 6F,G).

## Discussion

In the current study, we demonstrated that artemisinin prevented neuronal cells from glutamate-induced injury via the activation of Akt pathway. Our study identified a novel neuroprotective effect of artemisinin suggesting artemisinin could be potential therapeutic drug for prevention of neurodegenerative disorders.

Anti-malarial effect of artemisinin has been well established. Recently, anti-tumor and anti-inflammation properties have been demonstrated in artemisinin (Zuo et al., 2016). In addition, Zheng et al. (2016) demonstrate that pretreatment with 25 μM artemisinin for 1 h has anti-oxidize effect in nitroprusside-induced oxidative insult in cortical neuron by activating ERK pathway. The hippocampus is one of the most vulnerable parts of brain susceptible to various pathological conditions (Wang et al., 2016). In the current study, we explored the effect of artemisinin on oxidative stress using a HT-22 glutamate model. We found that pretreatment with 25 μM artemisinin for 12 h could provide HT-22 cells protection against glutamate-induced injury. However, pretreatment less than 12 h failed to protect HT-22 cells from glutamate toxicity. This seems to be a long preprocessing time to pretreat the cell for 12 h. There are several reasons for this phenomenon. First, it has been demonstrated that artemisinin exhibits time-dependent pharmacokinetics (Ashton et al., 1998; Gordi et al., 2002). Short pretreatment period of artemisinin may be insufficient and does not take effect (Zheng et al., 2016). Second, the dose which was used in our study to activate Akt enzymatic activity is much lower than preview study (Steely et al., 2017). Third, dihydroartemisinin is the active metabolite of all artemisinin compounds (Zhao et al., 2012). The metabolic processes of artemisinin to dihydroartemisinin may also contribute long preprocessing time to take effect.

Mitochondria is the main site for ROS production (Wang et al., 2016). We found that pretreatment with artemisinin decreased the subsequent glutamate-induced increase of mitochondrial ROS and total intracellular ROS levels. Accordingly, pretreatment with artemisinin attenuated the glutamate induced mitochondrial membrane potential collapse and rescued HT-22 cells form apoptotic cell death. It has been shown that prior treatment with mild ROS generatros may upregulate expression of hypoxia-inducible factor (HIF) and erythropoietin (Epo), and therefore protect neurons against subsequent ROS stress (Liu et al., 2005). Pretreatment with artemisinin may react with intracellular heme and elicit low levels production of ROS, which could protect cells from lethal ROS insult induced by glutamate damage (Schmuck et al., 2002; Kavishe et al., 2017).

Activation of Akt/Bcl-2 pathway has been demonstrated as an essential anti-apoptotic signaling (Ryou et al., 2013; Cao et al., 2017). The increased Bcl-2/Bax ratio blocks the cytochrome C released from mitochondria, which inhibits the mitochondrial apoptotic pathways (Zhu et al., 2016; Chauhan et al., 2018). The Akt signaling pathway has been indicated as an important drug target of artemisinin (Huang et al., 2013; Ho et al., 2014). The anti-cancer and anti-inflammatory effects of artemisinin have been attributed to the inhibition of Akt signaling pathway (Ho et al., 2014; Luo et al., 2015; Shao et al., 2017). On the other hand, Lee et al. (2012) observed that artemisinin could reduce inflammatory responses in microglial BV2 cells through activation of Akt signaling. In addition, Wang et al. (2015) reported that artemisinin could activate Akt signaling and trigger mitochondrial biogenesis in mice. In the current study, we found that Akt anti-apoptotic pathway was activated by artemisinin in a time dependent manner. Consistently, up-regulation of Bcl-2 and reduction of Bax, cleaved caspase-3 and cleaved PARP, downstream of Akt activation, were observed upon pretreatment of artemisinin. Furthermore, the protective effect of artemisinin was blocked by MK2206, a highly selective inhibitor of Akt, supporting that activation of Akt pathway was involved in the neuroprotective action of artemisinin.

**Figure 7:**
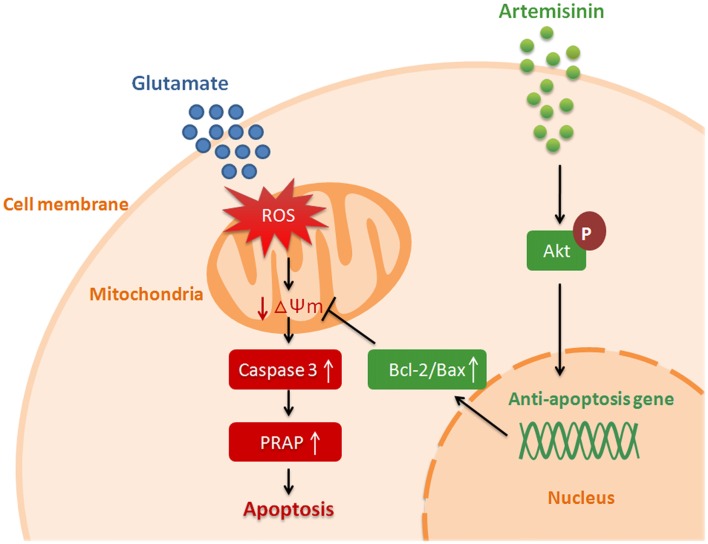
Hypothetical model of artemisinin mediated neuroprotection against glutamate-induced oxidative stress injury.

In summary, our results demonstrated that artemisinin protect neuronal HT-22 cell from glutamate-induced oxidative injury by activation of Akt signaling pathway (Figure 7). Due to its lipid-soluble characteristic, artemisinin can pass BBB and maintain a higher concentration in the central neural system (Zuo et al., 2016). As an FDA approval anti-malaria drug, artemisinin has been used in clinic for long-term without apparent adverse effects (Karbwang et al., 1992). Our finding indicates that artemisinin might be a potential novel antioxidant drug for the prevention and treatment of neurodegenerative disorders.

## Author Contributions

S-HY designed the experiments. S-PL, WL, AW and RL conducted the experiments. S-PL and S-HY wrote the manuscript. All the authors analyzed the data, revised the manuscript and approved the final manuscript.

## Conflict of Interest Statement

The authors declare that the research was conducted in the absence of any commercial or financial relationships that could be construed as a potential conflict of interest.
